# Using Machine Learning with Impulse Oscillometry Data to Develop a Predictive Model for Chronic Obstructive Pulmonary Disease and Asthma

**DOI:** 10.3390/jpm14040398

**Published:** 2024-04-09

**Authors:** Chien-Hua Huang, Kun-Ta Chou, Diahn-Warng Perng, Yi-Han Hsiao, Chien-Wen Huang

**Affiliations:** 1 Department of Eldercare, College of Nursing, Central Taiwan University of Science and Technology, Taichung 406053, Taiwan; 050205@tool.caaumed.org.tw; 2 Department of Chest Medicine, Taipei Veterans General Hospital, Taipei 112201, Taiwan; hbjoue@vghtpe.gov.tw (K.-T.C.); dwperng@vghtpe.gov.tw (D.-W.P.); yhhsiao2@vghtpe.gov.tw (Y.-H.H.); 3 Faculty of Medicine, School of Medicine, National Yang-Ming Chiao Tung University, Taipei 112304, Taiwan; 4 Division of Chest Medicine, Department of Internal Medicine, Asia University Hospital, Taichung 413505, Taiwan; 5 Department of Medical Laboratory Science and Biotechnology, College of Medical and Health Science, Asia University, Taichung 413305, Taiwan

**Keywords:** COPD, impulse oscillometry, machine learning

## Abstract

We aimed to develop and validate a machine learning model using impulse oscillometry system (IOS) profiles for accurately classifying patients into three assessment-based categories: no airflow obstruction, asthma, and chronic obstructive pulmonary disease (COPD). Our research questions were as follows: (1) Can machine learning methods accurately classify obstructive disease states based solely on multidimensional IOS data? (2) Which IOS parameters and modeling algorithms provide the best discrimination? We used data for 480 patients (240 with COPD and 240 with asthma) and 84 healthy individuals for training. Physiological and IOS parameters were combined into six feature combinations. The classification algorithms tested were logistic regression, random forest, neural network, k-nearest neighbor, and support vector machine. The optimal feature combination for identifying individuals without pulmonary obstruction, with asthma, or with COPD included 15 IOS and physiological features. The neural network classifier achieved the highest accuracy (0.786). For discriminating between healthy and unhealthy individuals, two combinations of twenty-three features performed best in the neural network algorithm (accuracy of 0.929). When distinguishing COPD from asthma, the best combination included 15 features and the neural network algorithm achieved an accuracy of 0.854. This study provides compelling technical evidence and clinical justifications for advancing IOS data-driven models to aid in COPD and asthma management.

## 1. Introduction

Artificial intelligence (AI) has significant applications in respiratory medicine. For example, it has been employed as a tool for evaluating chest computed tomography (CT) scans for lung cancer diagnosis [1]. In addition, a deep learning algorithm has been used for automated classification of fibrotic lung diseases in high-resolution CT scans [2]. This deep-learning algorithm, when applied to high-resolution CT scans, has the potential to offer cost-effective, reproducible, nearly instantaneous classification of fibrotic lung diseases with accuracy levels comparable to those of human experts. This holds particular promise for medical centers where thoracic imaging expertise is scarce [2].

Hwang et al. [3] have developed a deep neural network (DNN) capable of recognizing lung cancer, tuberculosis, pneumonia, and pneumothorax in chest radiographs. It also provides visual localization of abnormal findings. In another development, Yates et al. [4] created a chest radiograph triage system that employs binary classification, which categorizes radiographs as “normal” or “abnormal”. Impressively, this system achieved a final model accuracy of 94.6% for the test dataset. Furthermore, Lu et al. [5] have designed a DNN that accurately predicts all-cause mortality over a 12-year follow-up period using only a single plain chest radiograph. This prediction remains robust even after adjusting for radiologists’ diagnostic findings and standard risk factors of mortality. Using cases from the National Lung Cancer Screening Trial, Ardila et al. [6] successfully trained a DNN to predict lung cancer risk based on current and previous chest CT scans. This DNN achieved an area under the receiver operating characteristic curve (AUC) of 0.944 for predicting biopsy-proven cancer in the test dataset. Impressively, the accuracy of this DNN surpassed that of six board-certified radiologists when only the current CT scan was available and matched that of radiologists when both current and previous CT scans were accessible for review.

Diagnosing respiratory conditions, including asthma and chronic obstructive pulmonary disease (COPD), entails a comprehensive approach, encompassing patient history, physical examinations, pulmonary function tests, and, occasionally, medical imaging such as X-ray, CT, and bronchoscopy. While airflow limitation is a shared characteristic of asthma and COPD, their definitions are not mutually exclusive. Furthermore, these conditions are inherently heterogeneous, leading to different prognoses and management strategies [7,8]. The accurate diagnosis and differentiation of obstructive lung diseases such as COPD and asthma remain challenging, particularly in primary care settings where access to spirometry may be limited [9,10,11,12,13,14,15,16,17].

Recent research has explored the potential of AI in aiding the diagnosis and differentiation of respiratory conditions. One notable study evaluated the accuracy and interrater variability of pulmonologists in interpreting full pulmonary function tests, comparing their performance with that of AI-based software previously developed and validated using a substantial dataset of over 1430 historical cases. The findings demonstrated that the AI-based algorithm outperformed pulmonologist-based diagnostic categorization in terms of both sensitivity and positive predictive value across eight disease groups [18]. These results suggest that AI and machine learning hold promise as innovative tools for developing diagnostic algorithms for various medical conditions, including respiratory diseases [19].

While spirometry is considered the gold standard for assessing airflow limitation, it has certain limitations, especially in specific patient populations such as children, the elderly, and those with neuromuscular or behavioral challenges [20]. To address these limitations, an alternative technique, oscillometry, has gained traction for monitoring lung function and offers promising outcomes.

Impulse oscillometry (IOS) is a straightforward and noninvasive technique that requires nothing more than a patient’s passive cooperation. This method offers a valuable means of assessing lung function by measuring both airway resistance and airway reactance [21]. By harnessing sound waves, IOS swiftly detects changes in the airways and requires only normal tidal breathing from the patient. It operates as a variant of the forced oscillation technique, employing pressure oscillations at a fixed frequency of 5 Hz, from which all other relevant frequencies are derived. Pressure and flow transducers work in tandem to gauge amplitude and phase differences, enabling the determination of respiratory system impedance [22].

Several studies have explored the utility of IOS in diagnosing and evaluating obstructive lung diseases like COPD and asthma. For instance, Kanda et al. [23] demonstrated that IOS parameters, such as respiratory resistance and reactance, could effectively differentiate between asthma and COPD patients. Similarly, Liu et al. [24] found that IOS-derived indices like respiratory resistance at 5 Hz (R5) and the difference between R5 and R20 (R5-R20) were significantly associated with the severity of airflow limitation in COPD patients. Moreover, Li et al. [25] reported that IOS parameters like R5, R20, and the reactance area (AX) were sensitive markers for detecting airway obstruction in asthma. While these studies highlight the potential of IOS in respiratory medicine, the integration of IOS data with advanced analytical techniques like machine learning remains an area of active research.

Therefore, the aim of this study was to use IOS output parameters to identify the optimal combination of feature values and the best-performing classifier to develop an airflow obstruction clinical diagnosis support system that will assist clinicians in accurately diagnosing and treating patients.

## 2. Materials and Methods

### 2.1. Participants

This study collected data from outpatients of the Department of Chest Medicine in a university-affiliated hospital in Taiwan, from 1 January 2018 to 31 December 2020. A total of 3077 patients underwent both spirometry and IOS testing.

The diagnosis of COPD and asthma in this study was established based on the guidelines set forth by the Global Initiative for Chronic Obstructive Lung Disease (GOLD) and the Global Initiative for Asthma (GINA), respectively. According to the GOLD guidelines [26], COPD was diagnosed in individuals with a post-bronchodilator forced expiratory volume in 1 s (FEV1) to forced vital capacity (FVC) ratio of less than 0.70, in the presence of respiratory symptoms such as dyspnea, chronic cough, or sputum production. The severity of airflow limitation was further graded based on the post-bronchodilator FEV1 as follows: GOLD 1 (Mild) with FEV1 ≥ 80% predicted, GOLD 2 (Moderate) with 50% ≤ FEV1 < 80% predicted, GOLD 3 (Severe) with 30% ≤ FEV1 < 50% predicted, and GOLD 4 (Very Severe) with FEV1 < 30% predicted.

For asthma, the diagnosis was made in accordance with the GINA guidelines [27], which require the presence of respiratory symptoms such as wheezing, shortness of breath, chest tightness, and cough, along with variable expiratory airflow limitation. Specifically, individuals were diagnosed with asthma if they exhibited an increase in FEV1 of more than 12% and 200 mL from baseline, either spontaneously or after bronchodilator administration, confirming the presence of reversible airflow obstruction.

The exclusion criteria were as follows:

1. Patients with other respiratory conditions or comorbidities that could significantly affect lung function, such as lung cancer, interstitial lung diseases, or severe respiratory infections.
2. Patients with incomplete or missing data from either spirometry or IOS testing.
3. Patients who were unable to perform the lung function tests adequately due to cognitive or physical limitations.
4. Healthy volunteers with a history of smoking or any known respiratory condition.

Informed consent was not obtained from individual participants in this study as it involved a retrospective analysis of de-identified data. The dataset used in this research underwent a thorough de-identification process that removed any direct identifiers such as names, addresses, and social security numbers. Each participant in the dataset was assigned a unique anonymous identifier to ensure anonymity. The study design and data access procedures were reviewed and approved by the Feng-Yuan Hospital Ethics Committee (IRB Approval Number: 110016). Importantly, due to the de-identified nature of the data, the study was conducted in compliance with privacy regulations, and the results presented in this paper are not traceable to individual participants, ensuring the utmost confidentiality and privacy of the subjects involved.

### 2.2. Methods

**Figure 1:**
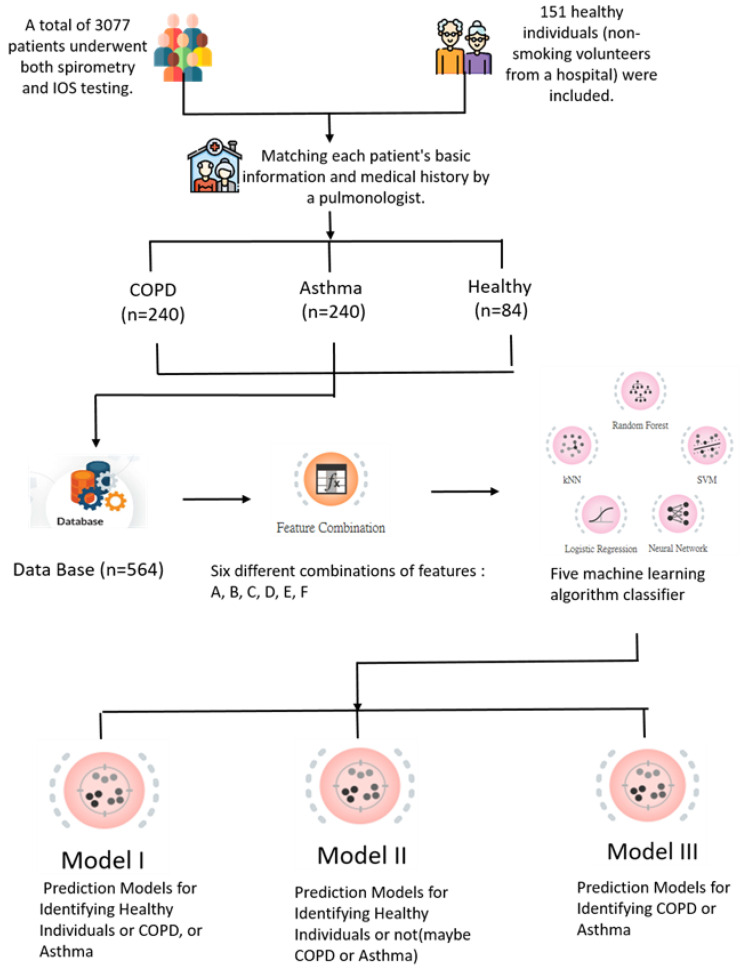
Flowchart showing the research steps.

This study referred to previous research [6,7,8,9,10] that suggested several machine learning algorithms suitable for exploring and analyzing data for the diagnosis of COPD and establishing classification prediction models. The classifiers used in this study included the following five: neural network using the Multiple Layer Perceptron (MLP) algorithm from sklearn; logistic regression (LR); random forest (RF); support vector machine (SVM); and k-nearest neighbor algorithm (KNN). This study employed the data mining and visual analysis software Orange3 (version 3.34.0) [28] for data analysis, feature embedding, building classification models, and predicting outcomes. It was based on IOS data and aimed to select suitable features to establish a predictive model for distinguishing between healthy individuals, COPD patients, and asthma patients. The research steps are shown in Figure 1.

### 2.3. Feature Combinations

Within the realm of IOS, resistance represents the in-phase component of lung impedance and offers insights into the forward pressure within conducting airways. In contrast, reactance constitutes the out-of-phase component of lung impedance, reflecting the capacitive and inertive characteristics of the airway. Capacitance may be likened to a reflection of airway elasticity, while inertance mirrors the mass inertial forces within the moving air column. Reactance can be visualized as rebound resistance, akin to an echo, supplying information about the distensible nature of the airway [29,30].

IOS testing provides a noninvasive and dynamic assessment of respiratory mechanics through measurements of resistance and reactance. As detailed above, parameters obtained from IOS offer valuable insights into total respiratory impedance along with central versus peripheral airway resistance. The frequency-dependent information captured by IOS allows characterization of optimal resonance properties and overall respiratory tissue mechanics. In particular, the reactance curve and its dependency on frequency reveals crucial details on the elastic and dynamic responses of the airways. Parameters such as the reactance area and resonant frequency facilitate the understanding of the interplay between capacitive and inertive forces governing airway function. In addition, the differences in resistance between low, high, and resonant frequencies enable site-specific assessment of small versus large airways.

Therefore, IOS parameters constitute information-rich, sensitive, and comprehensive indicators of respiratory status. The multidimensional data obtained from noninvasive IOS testing facilitates the capture of the intricacies and complex dynamics of the respiratory system. This wealth of embedded knowledge makes IOS parameters extremely well suited to the building of data-driven prediction models. The availability of resistance and reactance signatures across various frequencies enables machine learning algorithms to learn associations and patterns key to respiratory disease diagnosis and classification. In summary, the noninvasive, sensitive, and comprehensive nature of IOS data provides an ideal substrate for developing robust predictive models for assisting in the diagnosis of conditions such as asthma and COPD.

The feature values collected in this study were obtained from three data sources. The first was IOS output data, including R5, R10, R15, R20, R25, R35, X5, X10, X15, X20, X25, X35, Z5, VT, Rc, Rp, Fres, Ax, and R5–R20: 19 parameters in total. R5 represents the total airway resistance, and R20 represents the central airway resistance. The normal value should be within 150% of the expected value; in healthy people, both are very close, meaning the peripheral airway resistance is very small, represented by R5-R20. The X value represents the sum of elastic resistance and inertial resistance in respiratory impedance. X5 represents the peripheral elastic resistance. The difference between the predicted value and the measured value is not more than 0.2 kPa/(L·s), which is normal. Fres represents the resonant frequency (i.e., resonance point), which indicates that elastic resistance and inertial resistance are equal. It is the most sensitive indicator in bronchial function examination, and Fres in normal people does not exceed 10 Hz. AX represents the total reactance, signifying the area under the curve across all frequencies ranging from 5 Hz to Fres. Z5Hz is a parameter in IOS testing, representing the impedance of the respiratory system, measured at a frequency of 5 Hz. Respiratory impedance is a complex parameter that includes resistance (R) and reactance (X), which respectively represent the resistance to airflow and the elastic properties of the airways [23,29,30].

Specifically, Z5Hz indicates the total impedance of the respiratory system in response to external vibrations at a frequency of 5 Hz. The significance of this value lies in assessing the overall condition of the airways, including both central and peripheral components. Typically, the numerical values of Z5Hz fall within a positive range and can be used to evaluate the ventilation and elastic properties of the respiratory system. Rc (central airway resistance) represents the resistance of the central airways, including the trachea and large bronchi. A higher Rc value suggests greater resistance in the central airways, which may indicate some issues with the central airways [25].

Rp (peripheral airway resistance) represents the resistance of the peripheral airways, reflecting the resistance in the small airways such as small bronchi and alveoli. A higher Rp value suggests greater resistance in the peripheral airways, which may indicate issues with small airways or peripheral airways [25].

The second data source comprised the physiological characteristics of the participants, including age, gender, height, weight, and four other items.

The third included four derived parameters from IOS data based on recommendations from previous studies: R20 actual/predicted ratio; X5 predicted–actual difference; and Rc–Rp and Rp–Rc. Rc–Rp is the difference between Rc and Rp, often referred to as Rc–Rp. It is used in IOS testing to evaluate the difference in resistance between different parts of the airways, including central and peripheral airways. When Rc–Rp is larger, it indicates that the resistance in the central airways is relatively higher compared to the resistance in the peripheral airways, which may suggest issues or narrowing in the central airways. Conversely, when Rc–Rp is smaller, it suggests that the difference in resistance between central and peripheral airways is smaller, indicating a more uniform condition of the respiratory system.

Different feature combinations were then assembled based on different rationales, ultimately resulting in six combinations (Table 1). Combination A included only the 19 IOS output parameters to evaluate whether IOS data alone could build a robust prediction model without other influences. Combination B combined the nineteen IOS items with the four physiological parameters, as clinicians incorporate both data types for COPD/asthma diagnosis. Combination C selected seven key IOS measures (R5, R20, X5, Z5, Fres, Ax, and R5–R20) cited in previous studies, supplemented by the four derived IOS parameters, to give a total of eleven features. Combination D added the four physiological variables to C. Combination E combined the nineteen IOS outputs with the four derived IOS features. Combination F included all twenty-seven features from the three data sources.

## 3. Results

### 3.1. Model Performance

After a pulmonologist matched each patient’s basic information to their medical history, the diagnosis of COPD and asthma was established based on the guidelines set forth by the Global Initiative for Chronic Obstructive Lung Disease (GOLD) and the Global Initiative for Asthma (GINA), respectively, as described in the Materials and Methods section. The study included a total of 564 participants. Of these, 240 were patients diagnosed with COPD, 240 were patients diagnosed with asthma, and 84 were healthy non-smoking volunteers from the hospital who served as controls. Of these data samples, 80% (452 samples) were used as the training set and the remaining 20% (112 samples) were later used as the test set to evaluate model accuracy. Three predictive models were developed and evaluated using impulse IOS data and machine learning algorithms for the classification of respiratory diseases such as COPD and asthma. The first model differentiated between healthy individuals and patients with chronic obstruction diseases. The second model identified individuals as either healthy or having a respiratory disease. The third model differentiated between COPD and asthma patients. Five supervised learning classifiers were tested: neural networks, KNN, random forest, logistic regression, and SVM. Each model was trained and tested across the six input feature combinations (A–F), allowing performance assessment with IOS outputs alone versus the addition of physiological parameters and derived IOS measures.

#### 3.1.1. Model I: Screening Healthy Volunteers and Patients with COPD and Asthma

For differentiating disease from health (Model I), the neural network classifier achieved the highest average multi-class accuracy of 72.4%, ranging from 66.1% (Combination A with only IOS outputs) to 78.6% (Combination D with selective IOS parameters and physiological data). The AUC metric similarly indicated the superiority of the neural network with over 86.7% accuracy on average. Compared to IOS outputs alone, the augmentation of features increased accuracy, confirming the clinical relevance of knowledge of the patient’s physiology for screening applications (see Table 2).

#### 3.1.2. Model II: Detecting Respiratory Abnormalities

Model II performed better overall in identifying healthy cases, with the neural network, again, being superior at 90.8% average accuracy. Combinations B and F attained over 92% accuracy by additionally capitalizing on age, gender, height, and weight measures. Logistic regression and SVM classifiers also achieved strong performance with over 87% accuracy when physiological covariates were present. This robustness demonstrates the potential of using IOS data and machine learning to detect respiratory abnormalities for triaging and referral (see Table 3).

#### 3.1.3. Model III: Diagnostic Differentiation between Asthma and COPD

Model III involved more challenging multi-class differentiation between the obstructive diseases COPD and asthma. The performance of the metrics was understandably lower compared to that of the previous screening applications but still achieved mean accuracies over 70% for identifying individual diseases using only noninvasive IOS data. The neural network and logistic regression classifiers were most effective, benefiting more from the feature sets augmented with physiological parameters and IOS derivations than did the KNN, random forest, and SVM models. This indicates greater learning capacity of certain algorithms for diagnostic classification from multidimensional inputs (see Table 4).

### 3.2. Feature Importance

**Figure 2:**
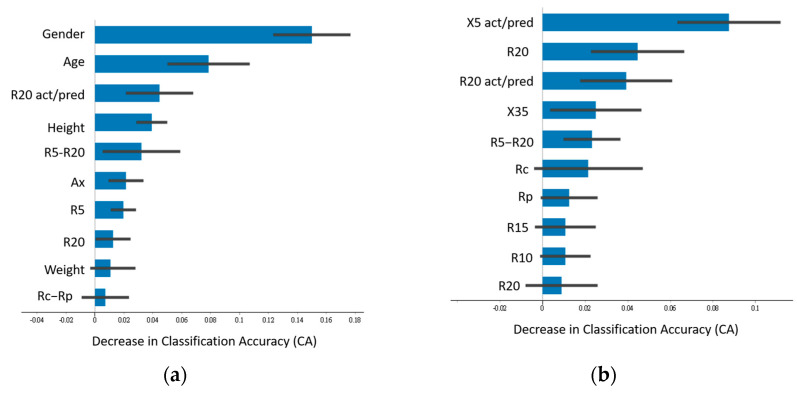
Ranking of feature importance of Model I. (**a**) Neural network classifier with feature Combination D. (**b**) Neural network classifier with feature Combination E.

**Figure 3:**
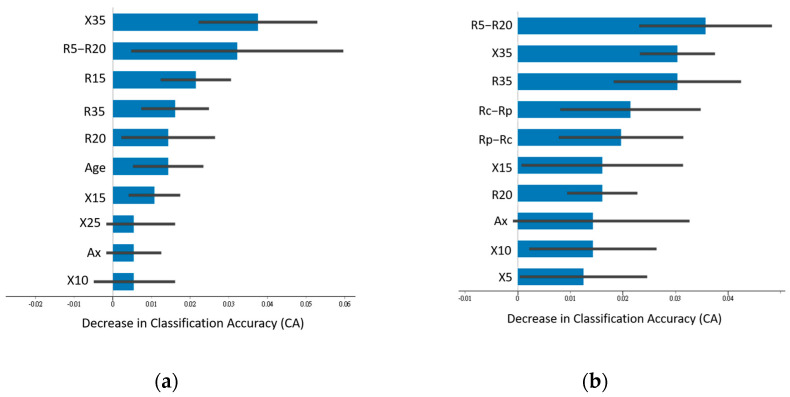
Ranking of feature importance of Model II. (**a**) Neural network classifier with feature Combination B. (**b**) Neural network classifier with feature Combination E.

**Figure 4:**
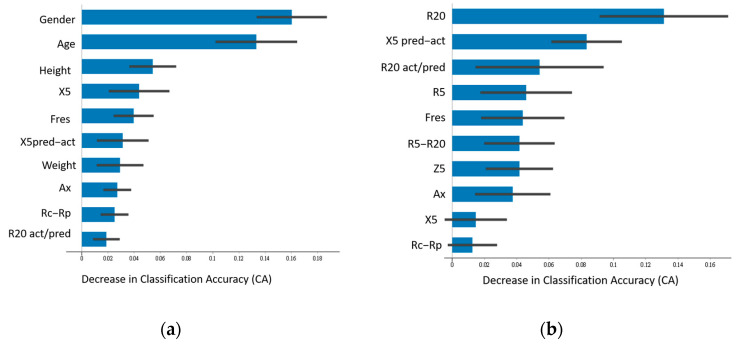
Ranking of feature importance of Model III. (**a**) Neural network classifier with feature Combination D. (**b**) Neural network classifier with feature Combination C.

Figure 2, Figure 3 and Figure 4 illustrate the top 10 most important features when the highest-performing neural network classifier was used with feature combinations with maximum accuracy.

For distinguishing disease status using Model I (Figure 2), the physiological variables gender, age, and height were the most impactful in Combination D (average accuracy of 0.755). Among IOS parameters, the actual-to-predicted resistance ratio at 20 Hz (R20 act/pred) and R5–R20 difference were influential (Figure 2a). Combination E (average accuracy of 0.623), which did not include physiological parameters, showed that reactance (X5) and resistors R20 and R20 act/pred were important. Irrespective of physiological parameters, R20 act/pred and R5–R20 emerged as key IOS features (Figure 2b).

In the identification of respiratory abnormalities (Model II; Figure 3), physiological parameters had minimal influence (except for age at rank 6 for Combination B, with an average accuracy of 0.893). Resistance measures R5–R20, R15, R20, and R35 and reactance X35 dominated feature importance (Figure 3a). Similar trends were seen for Combination E (average accuracy of 0.879), with R5–R20, R35, and X35 as the top features (Figure 3b).

In differentiating between COPD and asthma (Model III; Figure 4), physiological parameters were again ranked at the top in Combination D (average accuracy of 0.814; Figure 4a). In the case of Combination C without physiological parameters (average accuracy of 0.679), resistance R20, reactance deviation X5 pred–act, and resistance ratio R20 act/pred were the most informative IOS measures (Figure 4b).

## 4. Discussion

This study makes a compelling case for the viability of using machine learning models with IOS data to enable accurate, noninvasive diagnosis and screening of chronic obstruction diseases. The breadth of experiments across supervised classification algorithms, disease targets, and input feature sets facilitates insightful technical and clinical analysis.

The ability to differentiate disease from health (Model II), with up to 92% accuracy and an AUC of over 0.9, supports the utility of IOS testing for large-scale community-based screening. This could facilitate early intervention, improve outcomes, and lower healthcare costs. The high negative predictive value is especially useful for ruling out disease. Consistent with the literature [31], the finding that the addition of basic age, gender, height, and weight variables boosts accuracy further confirms the value of the physiological context. Standardization of robust screening approaches can aid adoption [32].

Importantly, the consistency with which low-frequency IOS resistance and reactance measures were identified as some of the most informative features closely reflects known respiratory physiology. Frequencies below 20 Hz enable greater penetration into the smaller distal airways. Higher resistance and more negative reactance values indicate increasing obstruction [29]. The prominence of resistance at 5 Hz (R5) and impedance (Z5) indicate their sensitivity, in agreement with the literature [23]. Moreover, the predictive value of derived indices, such as the R5–R20 difference and resistance ratios at differing frequencies, aligns with their ability to characterize site-specific mechanics and frequency dependence [23,29].

This embedded knowledge of physiology drives the gains in performance of the models compared to the performance of naive IOS data alone. The fact that differences in reactance values at 5 Hz compared to predictions (X5 pred–act) and resistance ratios relative to predicted normals (R20 act/pred) repeatedly arose as key features indicates the power of comparative measures over absolute values. The relative resistance between central and peripheral airways (Rc–Rp) also emerged as informative, reaffirming that the partitioning of respiratory impedance can benefit diagnosis [23,29]. The more surprising finding is that R35 and X35 play a highly important role in identifying healthy people or people with respiratory diseases. These two features have not been mentioned in previous studies, so further research is needed to verify their roles.

Among the classification algorithms, neural networks consistently emerged as superior performers across target conditions and feature sets owing to their greater learning capacity, aligning with trends in other medical applications [33]. The accuracy levels achieved by highly interpretable models such as SVM and logistic regression also make these models worthy of further optimization for clinical acceptance. The testing of model ensembles could reveal complementary advantages.

Among input features, Combinations B and D that contained a select subset of IOS output parameters augmented by physiological variables showed optimum trade-offs between predictive performance and parsimony. The drop in accuracy due to the elimination of lower-ranked IOS measures is modest, suggesting the potential to refine feature sets for generalizable models. The inclusion of additional variables, such as smoking history, imaging markers, and multi-omics profiles, could provide additive value.

In summary, this study provides convincing technical evidence, alongside physiological and clinical justifications, that strongly supports the value of advancing IOS data-driven models to aid COPD and asthma management. Standardization, prospective evaluation, and real-world validation of performance will be pivotal next steps toward understanding the translational impact of these models.

## 5. Conclusions

The experimental results of this study clearly demonstrate the feasibility of exploiting machine learning with IOS measures to establish robust models for both screening and diagnostic classification of common respiratory diseases. The integrative modeling approach provided clinically significant levels of accuracy by effectively combining noninvasive lung function profiles with relevant physiological knowledge. Areas for ongoing research include expanding disease groups beyond COPD and asthma, incorporating additional physiological data, and deploying model ensembles for boosted predictive performance. Prospective clinical validation can establish the viability of machine learning approaches as an assistive methodology to aid physicians’ expertise during respiratory diagnoses involving pulmonary function testing. While physiological data were useful for screening models in this study, IOS resistance and reactance parameters at low frequencies consistently emerged as key features across all target outcomes, reaffirming their relevance for diagnosing respiratory diseases.

## 6. Limitation

In this study, the diagnosis of COPD and asthma relied on spirometry and various factors, including smoking history, occupational exposure, atopy, age of onset, childhood asthma, and adherence to GOLD and GINA guidelines, respectively. These diagnoses were established by experienced pulmonologists following established guidelines, which encompassed a thorough assessment comprising spirometry results, patient history, and clinical presentation.

However, to narrow the focus of our study to exploring the effectiveness of machine learning techniques with IOS data, we excluded other pertinent factors such as smoking history, occupational exposure, atopy, age at onset, and childhood asthma from our predictive model. Incorporating these variables could enhance the accuracy and robustness of models designed to distinguish between COPD and asthma. Therefore, in future research endeavors, we intend to investigate the integration of these additional factors alongside IOS parameters to develop a more comprehensive predictive model. Such an approach has the potential to enhance the clinical utility of these models in aiding accurate diagnosis and management of respiratory diseases.

## Figures and Tables

**Table 1:** Six combinations of features in the study.

Combination	IOS Data (N = 19)	Selected IOS Data (N = 7)	Physiological Data (N = 4)	Conversion of IOS Data (N = 4)	Total
A	v				19
B	v		v		23
C		v		v	11
D		v	v	v	15
E	v			v	23
F	v		v	v	27

Note: A total of 19 features were extracted from the IOS output data. Of these, seven features—R5, R20, X5, Z5, Fres, Ax, and R5–R20—were considered crucial for the diagnosis of COPD based on previous studies. N = numbers of features. v = data source included in the features combination.

**Table 2:** Performance of prediction Model I for identifying healthy individuals, COPD patients, or asthma patients.

Feature Combination A					
Classifier	AUC (95%C.I)	CA	F1	Precision	Recall
MLP	0.815 (0.747, 0.883)	0.661	0.661	0.661	0.661
KNN	0.778 (0.706, 0.805)	0.571	0.571	0.574	0.571
RF	0.751 (0.675, 0.827)	0.527	0.504	0.528	0.527
LR	0.771 (0.698, 0.844)	0.571	0.566	0.567	0.571
SVM	0.758 (0.683, 0.833)	0.607	0.602	0.605	0.607
Feature Combination B					
Classifier	AUC (95%C.I)	CA	F1	Precision	Recall
MLP	0.924 (0.889, 0.959)	0.777	0.777	0.777	0.777
KNN	0.828 (0.762, 0.894)	0.714	0.709	0.716	0.714
RF	0.861 (0.803, 0.919)	0.661	0.643	0.649	0.661
LR	0.912 (0.872, 0.952)	0.750	0.726	0.743	0.750
SVM	0.917 (0.880, 0.954)	0.786	0.778	0.795	0.786
Feature Combination C					
Classifier	AUC (95%C.I)	CA	F1	Precision	Recall
MLP	0.822 (0.756, 0.888)	0.670	0.670	0.673	0.670
KNN	0.736 (0.663, 0.810)	0.607	0.599	0.620	0.607
RF	0.735 (0.662, 0.809)	0.545	0.539	0.647	0.545
LR	0.783 (0.714, 0.852)	0.625	0.617	0.615	0.625
SVM	0.738 (0.665, 0.811)	0.527	0.507	0.535	0.527
Feature Combination D					
Classifier	AUC (95%C.I)	CA	F1	Precision	Recall
MLP	0.911 (0.872, 0.950)	0.786	0.783	0.792	0.786
KNN	0.863 (0.805, 0.921)	0.750	0.747	0.749	0.750
RF	0.867 (0.811, 0.923)	0.714	0.685	0.719	0.714
LR	0.901 (0.859, 0.943)	0.750	0.727	0.732	0.750
SVM	0.898 (0.855, 0.941)	0.777	0.769	0.774	0.777
Feature Combination E					
Classifier	AUC (95%C.I)	CA	F1	Precision	Recall
MLP	0.818 (0.750, 0.886)	0.679	0.678	0.683	0.679
KNN	0.761 (0.686, 0.836)	0.607	0.606	0.620	0.607
RF	0.760 (0.685, 0.835)	0.527	0.509	0.521	0.527
LR	0.791 (0.723, 0.859)	0.625	0.621	0.621	0.625
SVM	0.803 (0.733, 0.873)	0.679	0.676	0.684	0.679
Feature Combination F					
Classifier	AUC (95%C.I)	CA	F1	Precision	Recall
MLP	0.909 (0.869, 0.949)	0.768	0.766	0.765	0.768
KNN	0.842 (0.780, 0.904)	0.705	0.702	0.706	0.705
RF	0.858 (0.800, 0.916)	0.661	0.643	0.654	0.661
LR	0.914 (0.875, 0.953)	0.759	0.740	0.755	0.759
SVM	0.909 (0.869, 0.949)	0.777	0.770	0.787	0.777

Note: CA refers to classification accuracy, which represents the proportion of correctly classified instances. Precision is the ratio of true positives to instances classified as positive. In this context, it represents the proportion of correctly identified “normal” cases among all instances classified as “normal.” Recall, also known as sensitivity or the true positive rate, is the ratio of true positives to all instances that are actually positive. In this context, it represents the proportion of truly “normal” individuals among all individuals identified as “normal”. F1 score is the weighted harmonic mean of precision and recall and provides a balanced measure of their combined performance.

**Table 3:** Performance of prediction Model II for identifying healthy individuals or patients with respiratory disease (COPD or asthma).

Feature Combination A					
Classifier	AUC (95%C.I)	CA	F1	Precision	Recall
MLP	0.953 (0.934, 0.972)	0.902	0.595	0.894	0.902
KNN	0.865 (0.809, 0.921)	0.875	0.872	0.869	0.875
RF	0.867 (0.811, 0.923)	0.866	0.838	0.845	0.866
LR	0.871 (0.816, 0.926)	0.866	0.829	0.852	0.866
SVM	0.931 (0.900, 0.962)	0.884	0.871	0.871	0.884
Feature Combination B					
Classifier	AUC (95%C.I)	CA	F1	Precision	Recall
MLP	0.942 (0.912, 0.972)	0.929	0.927	0.926	0.929
KNN	0.819 (0.752, 0.886)	0.893	0.883	0.883	0.893
RF	0.850 (0.789, 0.911)	0.866	0.829	0.852	0.866
LR	0.894 (0.852, 0.936)	0.866	0.829	0.852	0.866
SVM	0.928 (0.897, 0.959)	0.911	0.899	0.909	0.911
Feature Combination C					
Classifier	AUC (95%C.I)	CA	F1	Precision	Recall
MLP	0.916 (0.879, 0.953)	0.893	0.883	0.883	0.893
KNN	0.795 (0.726, 0.864)	0.875	0.868	0.865	0.875
RF	0.824 (0.757, 0.891)	0.857	0.831	0.830	0.857
LR	0.843 (0.781, 0.905)	0.839	0.789	0.774	0.839
SVM	0.869 (0.813, 0.925)	0.848	0.816	0.812	0.848
Feature Combination D					
Classifier	AUC (95%C.I)	CA	F1	Precision	Recall
MLP	0.906 (0.866, 0.946)	0.893	0.883	0.883	0.893
KNN	0.874 (0.819, 0.930)	0.884	0.882	0.881	0.884
RF	0.828 (0.761, 0.895)	0.857	0.812	0.833	0.857
LR	0.876 (0.821, 0.931)	0.848	0.794	0.801	0.848
SVM	0.880 (0.826, 0.934)	0.884	0.871	0.871	0.884
Feature Combination E					
Classifier	AUC (95%C.I)	CA	F1	Precision	Recall
MLP	0.957 (0.938, 0.976)	0.911	0.908	0.907	0.911
KNN	0.861 (0.805, 0.917)	0.893	0.887	0.885	0.893
RF	0.859 (0.802, 0.916)	0.848	0.816	0.812	0.848
LR	0.890 (0.847, 0.933)	0.857	0.812	0.833	0.857
SVM	0.928 (0.897, 0.959)	0.884	0.871	0.871	0.884
Feature Combination F					
Classifier	AUC (95%C.I)	CA	F1	Precision	Recall
MLP	0.946 (0.917, 0.975)	0.920	0.916	0.916	0.920
KNN	0.821 (0.754, 0.888)	0.893	0.887	0.885	0.893
RF	0.863 (0.806, 0.920)	0.866	0.829	0.852	0.866
LR	0.901 (0.859, 0.943)	0.866	0.829	0.852	0.866
SVM	0.931 (0.900, 0.962)	0.911	0.899	0.909	0.911

**Table 4:** Performance of prediction Model III for identifying COPD patients or asthma patients.

Feature Combination A					
Classifier	AUC (95%C.I)	CA	F1	Precision	Recall
MLP	0.751 (0.674, 0.828)	0.646	0.644	0.657	0.646
KNN	0.635 (0.552, 0.718)	0.573	0.569	0.586	0.573
RF	0.755 (0.679, 0.831)	0.667	0.665	0.678	0.667
LR	0.762 (0.686, 0.838)	0.688	0.686	0.700	0.688
SVM	0.619 (0.535, 0.703)	0.635	0.636	0.637	0.635
Feature Combination B					
Classifier	AUC (95%C.I)	CA	F1	Precision	Recall
MLP	0.902 (0.863, 0.941)	0.833	0.833	0.844	0.833
KNN	0.851 (0.790, 0.912)	0.802	0.801	0.821	0.802
RF	0.883 (0.830, 0.936)	0.771	0.767	0.808	0.771
LR	0.902 (0.863, 0.941)	0.802	0.800	0.829	0.802
SVM	0.897 (0.854, 0.940)	0.823	0.822	0.843	0.823
Feature Combination C					
Classifier	AUC (95%C.I)	CA	F1	Precision	Recall
MLP	0.763 (0.688, 0.838)	0.698	0.693	0.725	0.698
KNN	0.685 (0.606, 0.764)	0.615	0.611	0.630	0.615
RF	0.723 (0.645, 0.801)	0.667	0.667	0.668	0.667
LR	0.750 (0.674, 0.826)	0.677	0.670	0.709	0.677
SVM	0.672 (0.591, 0.753)	0.625	0.624	0.624	0.625
Feature Combination D					
Classifier	AUC (95%C.I)	CA	F1	Precision	Recall
MLP	0.899 (0.858, 0.940)	0.854	0.854	0.861	0.854
KNN	0.865 (0.809, 0.921)	0.802	0.802	0.807	0.802
RF	0.869 (0.813, 0.925)	0.781	0.779	0.807	0.781
LR	0.900 (0.860, 0.940)	0.812	0.811	0.836	0.812
SVM	0.901 (0.861, 0.941)	0.823	0.822	0.837	0.823
Feature Combination E					
Classifier	AUC (95%C.I)	CA	F1	Precision	Recall
MLP	0.753 (0.677, 0.829)	0.677	0.672	0.702	0.677
KNN	0.695 (0.616, 0.774)	0.646	0.640	0.671	0.646
RF	0.762 (0.686, 0.838)	0.688	0.685	0.705	0.688
LR	0.778 (0.704, 0.852)	0.688	0.680	0.725	0.688
SVM	0.703 (0.623, 0.783)	0.698	0.693	0.725	0.698
Feature Combination F					
Classifier	AUC (95%C.I)	CA	F1	Precision	Recall
MLP	0.890 (0.846, 0.934)	0.854	0.854	0.866	0.854
KNN	0.866 (0.810, 0.922)	0.792	0.791	0.807	0.792
RF	0.881 (0.828, 0.934)	0.781	0.778	0.815	0.781
LR	0.902 (0.863, 0.941)	0.802	0.800	0.829	0.802
SVM	0.899 (0.858, 0.940)	0.812	0.812	0.829	0.812

## Data Availability

The data presented in this study are available on request from the corresponding author. The data are not publicly available due to it belonging to individuals who participated in the study.
